# Supplementary material for: Aneuploidy tolerance caused by BRG1 loss allows chromosome gains and recovery of fitness
Source: Nat Commun. 2022 Apr 1;13:1731. doi: 10.1038/s41467-022-29420-3 (PMC8975814; doi:10.1038/s41467-022-29420-3)
Supplement: Supplementary file 1 — Supplementary Information [file 41467_2022_29420_MOESM1_ESM.pdf]

Supplementary Information

Aneuploidy tolerance caused by BRG1 loss allows chromosome gains and recovery of fitness

Supplementary Table 1. Relationship between BRG1 (SMARCA4) expression and expression levels of immunoproteasome and PA28 subunits in cancer samples. Correlation between mRNA expression levels of BRG1 (SMARCA4) and the indicated gene were analysed using Spearman's rank order correlation coefficient from cBioPortal data for colorectal adenocarcinoma (COAD, TCGA Firehose legacy, n=379) and for lung adenocarcinoma (LUAD, TCGA Firehose legacy, n=230). BRG1 expression is inversely correlated with immunoproteasome and PA28 complex gene expression in lung cancer but not colorectal cancer samples. The p values were derived using a 2-sided t-test.

	**COAD**		**LUAD**	
**Gene**	*Spearman coefficient*	*p value*	*Spearman coefficient*	*p value*
PSMB8	-0.912	0.075	-0.298	4.096e-6
PSMB9	-0.024	0.635	-0.190	3.126e-3
PSMB10	-0.505	0.324	-0.175	7.729e-3
PSME1	-0.190	0.0336	-0.320	6.40e-7
PSME2	3.1e-3	0.952	-0.236	3.14e-4

Supplementary Table 2. Oligonucleotides used in this study

siBRG1 1 (5′ GGGUACCCUCAGGACAACA 3')	Eurofins
siBRG1 2 (5′ CGACGUACGAGUACAUCAU 3')	Eurofins
siBRG1 5 (5' GCACACCGCUGCAGAACAA 3')	Dharmacon
siBRG1 6 (5' CCAAGCCGGUCGUGAGUGA 3')	Dharmacon
siBRG1 7 (5' GCGACUCACUGACGGAGAA 3')	Dharmacon
siBRG1 8 (5' GACCAGCACUCCCAAGGUU 3')	Dharmacon
ON-TARGET plus Non-targeting Pool	Dharmacon
sgRNA sequence targeting BRG1 (5' caccgGGCCGAGGAGTTCCGCCCAG 3', 5' aaacCTGGGCGGAACTCCTCGGCCc 3')	This paper
PSMB8 fwd (5' GATGGACCCCGTGGAAAGAT 3')	This paper
PSMB8 rev (5' ACATGGTGCCAAGCAGGTAA 3')	This paper
PSMB9 fwd (5' TGTGATGGGTTCTGATTCCCG 3')	This paper
PSMB9 rev (5' GAGTGCACAGTAGATGCGCT 3')	This paper
PSMB10 fwd (5' ATACGCGAGCCACTAACGATT 3')	This paper
PSMB10 rev (5' GGTTCCAGGCACAAAGTGGT 3')	This paper
PSME1 fwd (5' TTTTGGAGTGGCTGTCCAGG 3')	This paper
PSME1 rev (5' AGCATTGCGGATCTCCATGA 3')	This paper
PSME2 fwd (5' GCTGACTTGACTTCCCTCCG 3')	This paper
PSME2 rev (5' GAGTCTCCTTGGAGGCCTTG 3')	This paper
STAT1 fwd (5' ATGGCAGTCTGGCGGCGGCTGAATT 3')	This paper
STAT1 rev (5' CCAAACCAGGCTGGCACAATTG 3')	This paper
STAT2 fwd (5' CAGGTCACAGAGTTGCTACAGC 3')	This paper
STAT2 rev (5' CGGTGAACTTGCTGCCAGTCTT 3')	This paper
GAPDH fwd (5' ACATCGCTCAGACACCATG 3')	Meisenberg, 2015
GAPDH rev (5' TGTAGTTGAGGTCAATGAAGGG 3')	Meisenberg, 2015

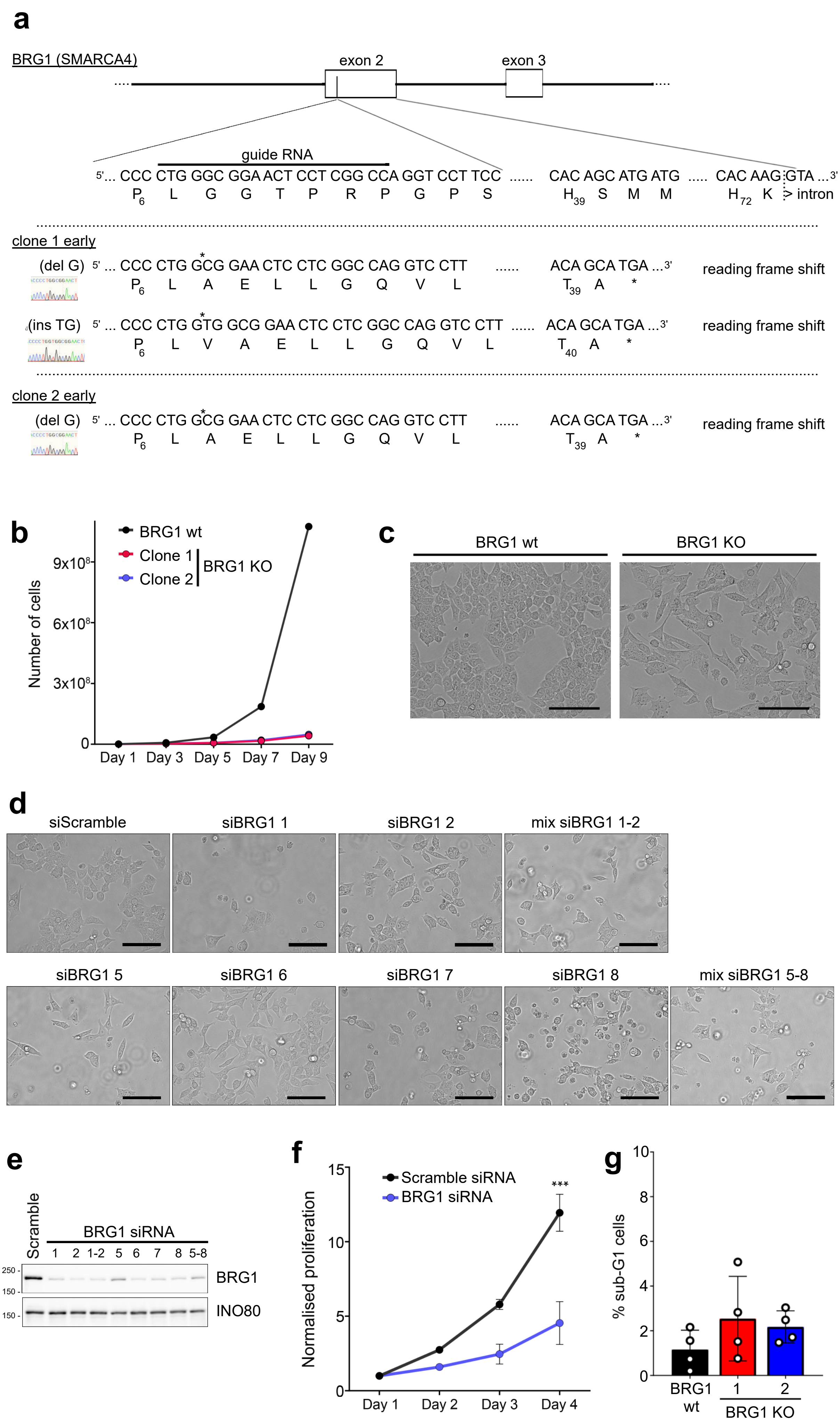
a
BRG1 (SMARCA4)
exon 2
exon 3
guide RNA
5'... CCC CTG GGC GGA ACT CCT CGG CCA GGT CCT TCC ...... CAC AGC ATG ATG ..... CAC AAG GTA ...3'
P6 L G G T P R P G P S H39 S M M H72 K > intron
clone 1 early
(del G) 5' ... CCC CTG GCG GAA CTC CTC GGC CAG GTC CTT ...... ACA GCA TGA ...3' reading frame shift
P6 L A E L L G Q V L T39 A *
(ins TG) 5' ... CCC CTG GTG GCG GAA CTC CTC GGC CAG GTC CTT ...... ACA GCA TGA ...3' reading frame shift
P6 L V A E L L G Q V L T40 A *
clone 2 early
(del G) 5' ... CCC CTG GCG GAA CTC CTC GGC CAG GTC CTT ...... ACA GCA TGA ...3' reading frame shift
P6 L A E L L G Q V L T39 A *
b
BRG1 wt
Clone 1
Clone 2
BRG1 KO
Number of cells
9x10⁸
6x10⁸
3x10⁸
0
Day 1 Day 3 Day 5 Day 7 Day 9
c
BRG1 wt
BRG1 KO
d
siScramble
siBRG1 1
siBRG1 2
mix siBRG1 1-2
siBRG1 5
siBRG1 6
siBRG1 7
siBRG1 8
mix siBRG1 5-8
e
Scramble
BRG1 siRNA
1 2 1-2 5 6 7 8 5-8
250
150
150
BRG1
INO80
f
Scramble siRNA
BRG1 siRNA
***
Normalised proliferation
15
10
5
0
Day 1 Day 2 Day 3 Day 4
g
% sub-G1 cells
10
8
6
4
2
0
BRG1 wt
1
2
BRG1 KO

Supplementary figure legends

Fig. S1 Morphology changes and loss of fitness are associated with loss of BRG1 in HCT116 cells. **a** Sequencing data for BRG1 knockout clones 1 and 2. Position of guide RNA is indicated. The changes in sequence and resulting alterations in protein coding are indicated. **b** Proliferation rate of HCT116 (BRG1 wt) and BRG1 knockout clones 1 and 2. Cell number was monitored every 2 days over a period of 9 days. n = 1. **c** Cellular morphology of the BRG1 knockout clone 2 shows differences with the parental HCT116 cell line (BRG1 wt). Scale bar = 100 µm. Similar results were obtained in three independent repeats. **d** Cellular morphology following siRNA depletion of BRG1 in HCT116 shows differences with HCT116 cells transfected with a non-targeting control sequence (siScramble). HCT116 cells were transfected with the indicated siRNA sequences targeting BRG1. Scale bar = 100 µm. Similar results were obtained in three independent repeats. **e** Western blot analysis of BRG1 in whole cell extracts prepared from HCT116 transfected with non-targeting control siRNA (siScramble) and the indicated siRNA targeting BRG1. INO80 was used as loading control. Similar results were obtained in at least two independent repeats for all siRNAs, and in more than three independent repeats for the Smartpool and siBRG1-6. **f** Normalised proliferation from four independent experiments obtained with CellTiterGlo Luminescent Cell Viability assay between HCT116 transfected with non-targeting control siRNA (siScramble) and siRNA targeting BRG1 (siBRG1 1-2). Data are presented as the mean ±SD; n = 4 independent biological experiments. The p-value was calculated with two-way ANOVA-Bonferroni. *** $p < 0.001$. **g** Quantification of sub-G1 cells in the parental HCT116 and BRG1 KO clones as measured by FACS (related to Fig. 1d). Data are represented as the mean ±SD; n = 4 independent biological experiments.

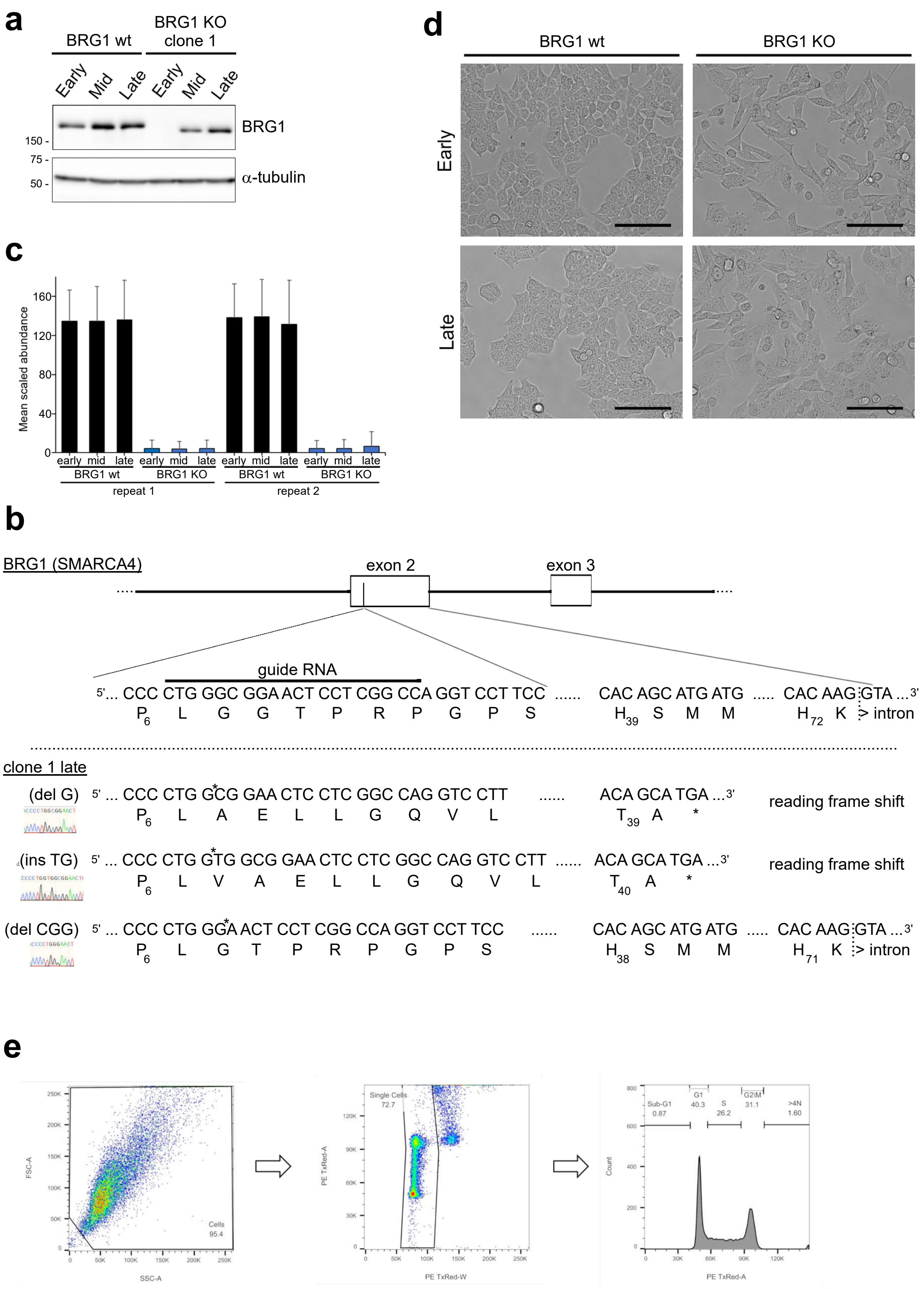
a
BRG1 wt
BRG1 KO clone 1
Early Mid Late Early Mid Late
BRG1
150 -
75 -
50 -
α-tubulin
c
Mean scaled abundance
160
120
80
40
0
early mid late early mid late early mid late early mid late
BRG1 wt BRG1 KO BRG1 wt BRG1 KO
repeat 1 repeat 2
d
BRG1 wt
BRG1 KO
Early
Late
b
BRG1 (SMARCA4)
exon 2
exon 3
guide RNA
5'... CCC CTG GGC GGA ACT CCT CGG CCA GGT CCT TCC ...... CAC AGC ATG ATG ..... CAC AAG GTA ...3'
P6 L G G T P R P G P S H39 S M M H72 K > intron
clone 1 late
(del G) 5' ... CCC CTG GCG GAA CTC CTC GGC CAG GTC CTT ...... ACA GCA TGA ...3' reading frame shift
P6 L A E L L G Q V L T39 A *
(ins TG) 5' ... CCC CTG GTG GCG GAA CTC CTC GGC CAG GTC CTT ...... ACA GCA TGA ...3' reading frame shift
P6 L V A E L L G Q V L T40 A *
(del CGG) 5' ... CCC CTG GGA ACT CCT CGG CCA GGT CCT TCC ...... CAC AGC ATG ATG ..... CAC AAG GTA ...3'
P6 L G T P R P G P S H38 S M M H71 K > intron
e
FSC-A
SSC-A
Cells 95.4
Single Cells 72.7
PE TxRed-A
PE TxRed-W
Count
Sub-G1 0.87
G1 40.3
S 26.2
G2\M 31.1
>4N 1.60

Fig. S2 BRG1 re-expression occurred in BRG1-deficient clone 1 but not clone 2 after long term cell culture. **a** Western blot analysis of BRG1 from whole cell extracts prepared from HCT116 (BRG1 wt) or BRG1 knockout cells (BRG1 KO clone 1) at early, mid (4 months), or late (8 months) time points. α-tubulin was used as loading control. Similar results were obtained in three independent repeats. **b** Sequencing data for BRG1 knockout clone 1 after 8 months in culture. Position of guide RNA is indicated. The changes in sequence and resulting protein coding alterations are indicated. **c** Analysis of data from two mass spectrometry repeats from HCT116 parental cells and BRG1 KO clone 2 at early and late time points. BRG1 KO clone 2 shows no evidence of BRG1 re-expression Analysis was performed using all 29 potential isoforms of BRG1 retrieved from Uniprot. Data are represented as the mean ±SD; n=29. **d** Morphology of BRG1 KO clone 2 cells changes during long term cell culture. Micrographs of HCT116 and BRG1 knockout cells at early and late (8 months) time points. Scale bar = 100 μm. Similar results were obtained in three independent repeats. **e.** Gating strategy for FACS analyses used in Figures 1 and 2. Individual events were first gated to remove events inconsistent with intact cells (left panel), then further gated to remove cellular aggregates (middle panel). Finally, cell cycle phase boundaries were set (right panel) as shown in Figures 1 and 2.

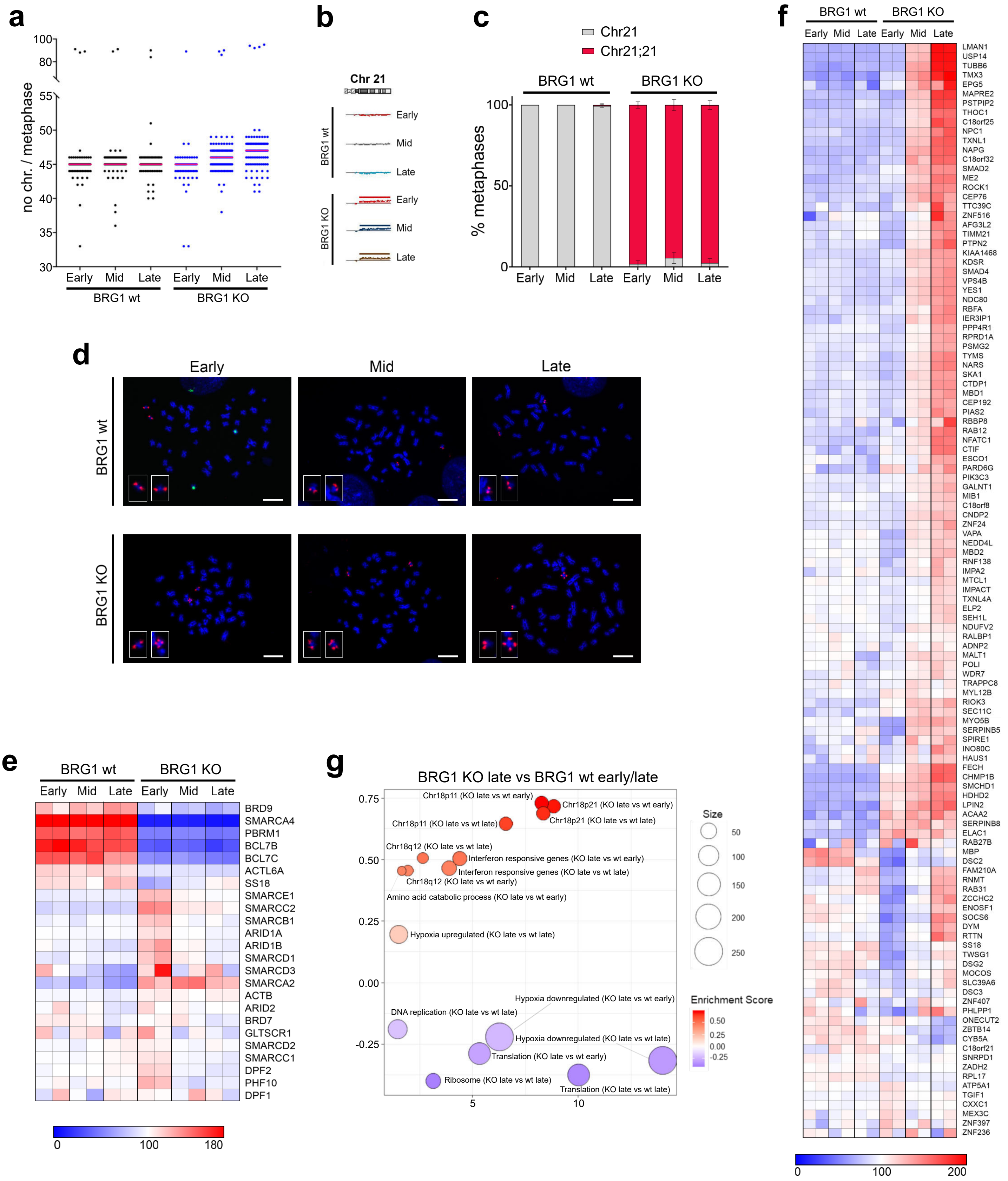
a
no chr. / metaphase
100
80
55
50
45
40
35
30
Early
Mid
Late
Early
Mid
Late
BRG1 wt
BRG1 KO
b
Chr 21
BRG1 wt
BRG1 KO
Early
Mid
Late
Early
Mid
Late
c
Chr21
Chr21;21
BRG1 wt
BRG1 KO
% metaphases
100
50
0
Early
Mid
Late
Early
Mid
Late
d
Early
Mid
Late
BRG1 wt
BRG1 KO
e
BRG1 wt
BRG1 KO
Early
Mid
Late
Early
Mid
Late
BRD9
SMARCA4
PBRM1
BCL7B
BCL7C
ACTL6A
SS18
SMARCE1
SMARCC2
SMARCB1
ARID1A
ARID1B
SMARCD1
SMARCD3
SMARCA2
ACTB
ARID2
BRD7
GLTSCR1
SMARCD2
SMARCC1
DPF2
PHF10
DPF1
0
100
180
g
BRG1 KO late vs BRG1 wt early/late
0.75
0.50
0.25
0.00
-0.25
5
10
Chr18p11 (KO late vs wt early)
Chr18p21 (KO late vs wt early)
Chr18p11 (KO late vs wt late)
Chr18p21 (KO late vs wt late)
Chr18q12 (KO late vs wt late)
Interferon responsive genes (KO late vs wt early)
Interferon responsive genes (KO late vs wt late)
Chr18q12 (KO late vs wt early)
Amino acid catabolic process (KO late vs wt early)
Hypoxia upregulated (KO late vs wt late)
Hypoxia downregulated (KO late vs wt early)
DNA replication (KO late vs wt late)
Hypoxia downregulated (KO late vs wt late)
Translation (KO late vs wt early)
Ribosome (KO late vs wt late)
Translation (KO late vs wt late)
Size
50
100
150
200
250
Enrichment Score
0.50
0.25
0.00
-0.25
f
BRG1 wt
BRG1 KO
Early
Mid
Late
Early
Mid
Late
LMAN1
USP14
TUBB6
TMX3
EPG5
MAPRE2
PSTPIP2
THOC1
C18orf25
NPC1
TXNL1
NAPG
C18orf32
SMAD2
ME2
ROCK1
CEP76
TTC39C
ZNF516
AFG3L2
TIMM21
PTPN2
KIAA1468
KDSR
SMAD4
VPS4B
YES1
NDC80
RBFA
IER3IP1
PPP4R1
RPRD1A
PSMG2
TYMS
NARS
SKA1
CTDP1
MBD1
CEP192
PIAS2
RBBP8
RAB12
NFATC1
CTIF
ESCO1
PARD6G
PIK3C3
GALNT1
MIB1
C18orf8
CNDP2
ZNF24
VAPA
NEDD4L
MBD2
RNF138
IMPA2
MTCL1
IMPACT
TXNL4A
ELP2
SEH1L
NDUFV2
RALBP1
ADNP2
MALT1
POLI
WDR7
TRAPPC8
MYL12B
RIOK3
SEC11C
MYO5B
SERPINB5
SPIRE1
INO80C
HAUS1
FECH
CHMP1B
SMCHD1
HDHD2
LPIN2
ACAA2
SERPINB8
ELAC1
RAB27B
MBP
DSC2
FAM210A
RNMT
RAB31
ZCCHC2
ENOSF1
SOCS6
DYM
RTTN
SS18
TWSG1
DSG2
MOCOS
SLC39A6
DSC3
ZNF407
PHLPP1
ONECUT2
ZBTB14
CYB5A
C18orf21
SNRPD1
ZADH2
RPL17
ATP5A1
TGIF1
CXXC1
MEX3C
ZNF397
ZNF236
0
100
200

Fig. S3 The BRG1 knockout cells (clone 2) have a chromosome 21 fusion and show proteomic changes consistent with loss of BRG1 and increasing chromosome 18 copy number. **a** Quantification of chromosome number per metaphase spread in parental HCT116 and BRG1 KO cells at early, mid and late time points. Each dot represents a single metaphase spread and the red line indicates the median. **b** CGH array data for chromosome 21 showing copy number variations (CNVs) for chromosome 21 at early, mid (4 months) and late (8 months) time points in the BRG1 knockout (BRG1 KO) compared with HCT116 (BRG1 wt). **c** Percentage of normal chromosome 21 and isochromosome 21 per metaphase in HCT116 (BRG1 wt) and BRG1-deficient cells (BRG1 KO) at early, mid and late time points. Data are presented as the mean ±SD; n = 3. **d** Representative metaphase FISH from HCT116 (BRG1 wt) and BRG1-deficient cells (BRG1 KO clone 2) at early, mid and late time points with a probe against chromosome 21 (red). The FISH signals indicate the presence of an isochromosome 21 in the BRG1 knockout cells. Similar results were obtained in three independent repeats. The insets (white boxes) show zoomed in chr21 signals. Scale bar = 10 μm. **e** Heatmap showing scaled abundance of SWI/SNF complex subunits detected by proteome analysis in HCT116 (BRG1 wt) and BRG1 knockout cells (BRG1 KO) at early, mid (4 months), and late (8 months) time points. Duplicates are shown side by side. The colour key indicates the relative abundance of each protein (scale: 0-180). **f** Heatmap showing scaled abundance of chromosome 18 proteins detected by proteome analysis in HCT116 (BRG1 wt) and BRG1 knockout cells (BRG1 KO) at early, mid (4 months), and late (8 months) time points. Duplicates are shown side by side. The colour key indicates the relative abundance of each protein (scale: 0-200). **g** Pathway enrichment analysis showing changes in BRG1-deficient (KO) cells at late time points compared with parental HCT116 cells. For the analysis, the proteome from BRG1-deficient cells at late (8 months) time points was compared to the proteome of HCT116 cells at all time points. Upregulated pathways (in red) and downregulated pathways (in blue) are shown according to the enrichment score. Dot size varies accordingly to the size of the pathway.

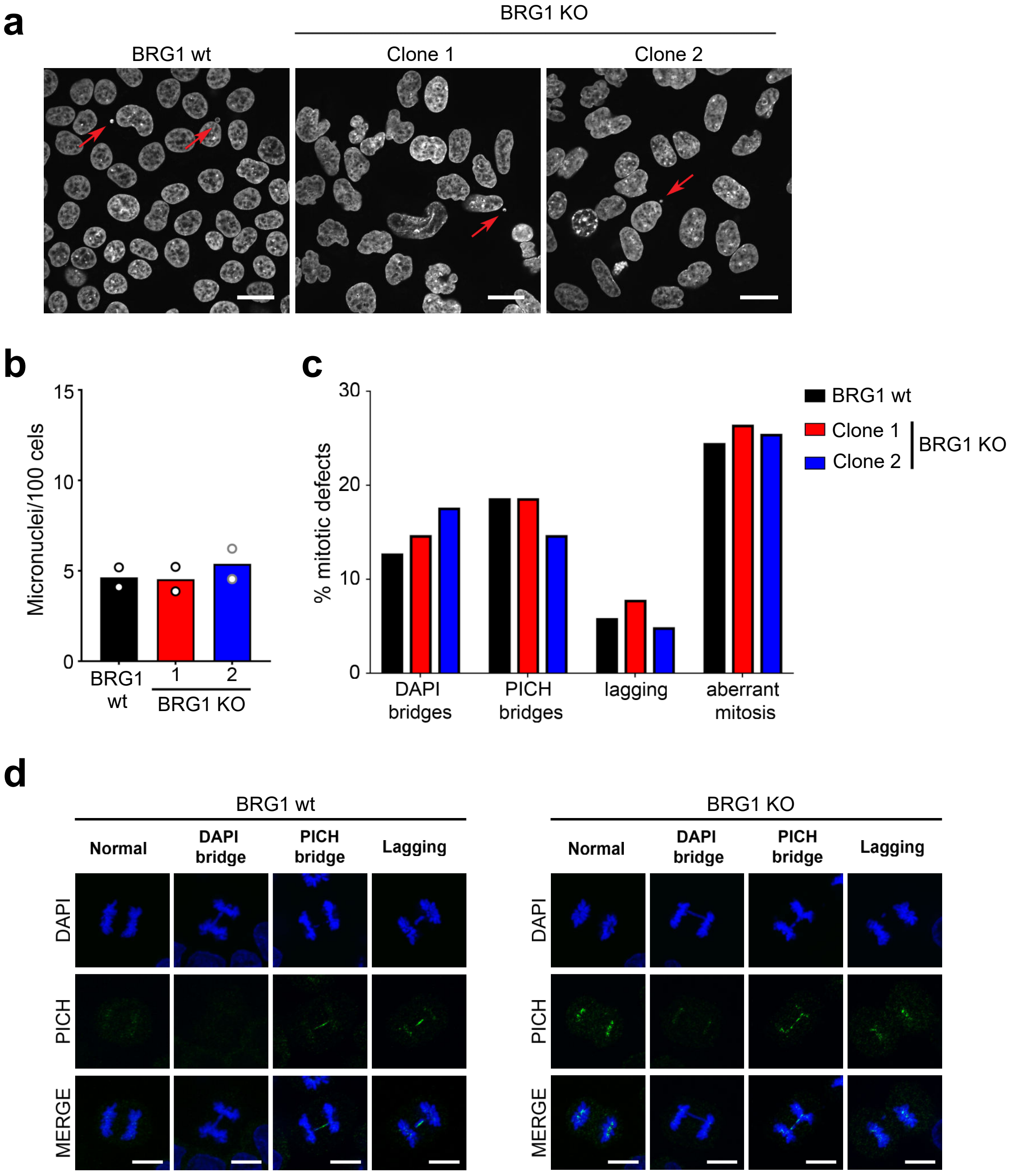
a
BRG1 KO
BRG1 wt
Clone 1
Clone 2
b
Micronuclei/100 cels
15
10
5
0
BRG1
wt
1
2
BRG1 KO
c
% mitotic defects
30
20
10
0
DAPI
bridges
PICH
bridges
lagging
aberrant
mitosis
BRG1 wt
Clone 1
Clone 2
BRG1 KO
d
BRG1 wt
BRG1 KO
Normal
DAPI
bridge
PICH
bridge
Lagging
DAPI
PICH
MERGE

Fig. S4 The BRG1 knockout cell lines don't show evidence of increased chromosomal instability. **a** Representative DAPI stained nuclei in HCT116 (BRG1 wt) and three BRG1 knockout clones at early time points. Red arrows indicate micronuclei. Scale bar = 20 µm. **b** Percentage of micronuclei in cells described in **a**. Micronuclei from at least 1000 cells were manually counted. Data are presented as the mean; n = 2. **c** Quantification of mitotic defects in HCT116 (BRG1 wt) cells and three BRG1-deficient HCT116 clones (BRG1 KO). DAPI bridges, PICH-positive bridges, and lagging chromosomes were analysed and plotted separately or as total aberrant mitosis events. n=1. **d** Representative images of mitotic defects observed in cells described in **c**. Scale bar = 10 µm.

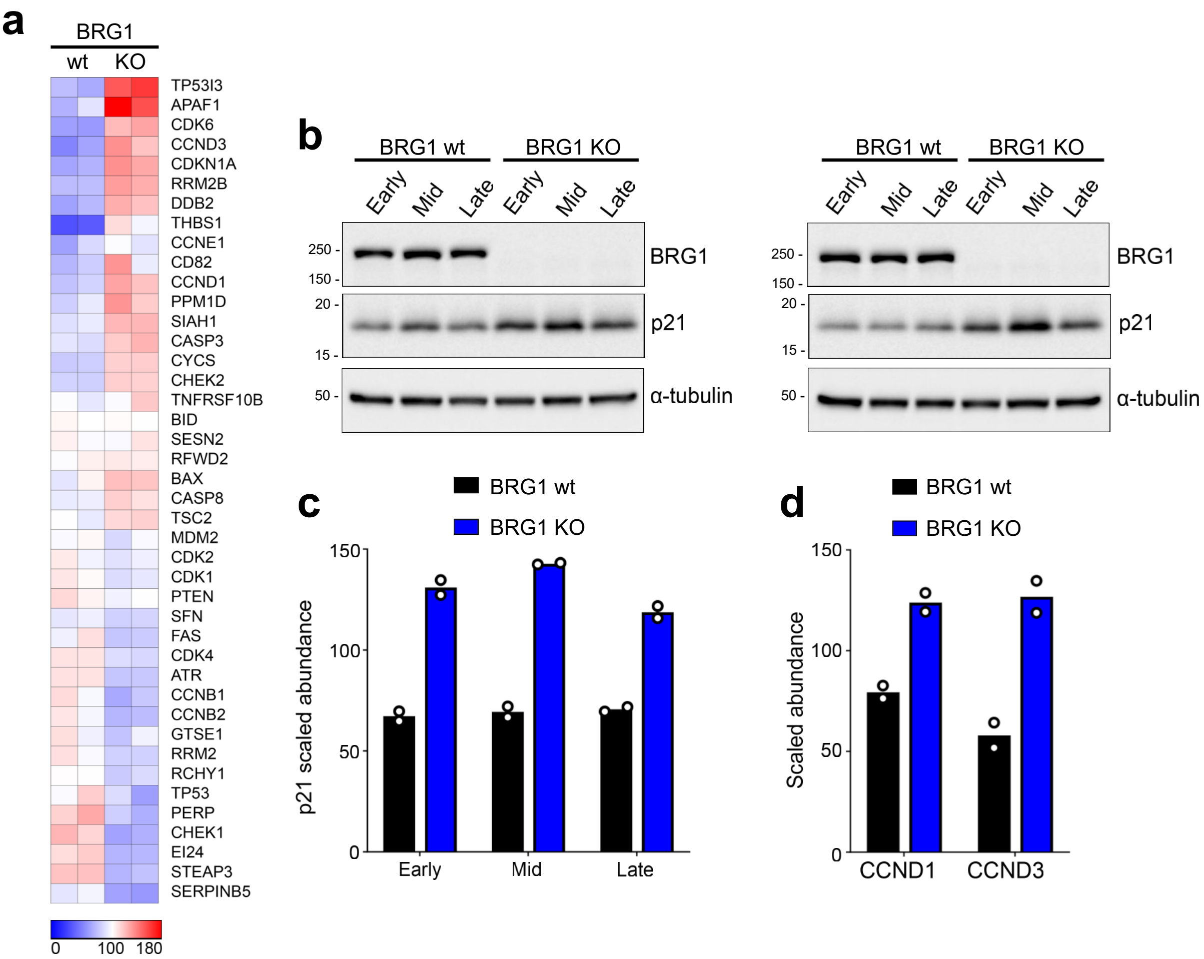
a
BRG1
wt
KO
TP53I3
APAF1
CDK6
CCND3
CDKN1A
RRM2B
DDB2
THBS1
CCNE1
CD82
CCND1
PPM1D
SIAH1
CASP3
CYCS
CHEK2
TNFRSF10B
BID
SESN2
RFWD2
BAX
CASP8
TSC2
MDM2
CDK2
CDK1
PTEN
SFN
FAS
CDK4
ATR
CCNB1
CCNB2
GTSE1
RRM2
RCHY1
TP53
PERP
CHEK1
EI24
STEAP3
SERPINB5
0
100
180
b
BRG1 wt
BRG1 KO
Early
Mid
Late
250 -
150 -
20 -
15 -
50 -
BRG1
p21
α-tubulin
c
BRG1 wt
BRG1 KO
p21 scaled abundance
150
100
50
0
Early
Mid
Late
d
Scaled abundance
CCND1
CCND3

Fig. S5 Misregulation of the p53 pathway is evident in the BRG1 knockout cells. **a** Heatmap showing scaled abundance of p53 pathway proteins (KEGG p53 signalling pathway) in HCT116 (BRG1 wt) and BRG1-deficient cells (BRG1 KO clone 2) at early time points. Duplicates are shown side by side. The colour key indicates the relative abundance of each protein (scale: 0-180). **b** p21 levels are consistently higher in the BRG1 knockout cells at all time points. Western blot analysis of p21 levels in whole cell extracts prepared from HCT116 (BRG1 wt) and BRG1 knockout cells (BRG1 KO) at early, mid (4 months) and late (8 months) time points. Each panel represents an independent experiment. α-tubulin was used as loading control. **c** Scaled abundance of p21 by proteome analysis in HCT116 (BRG1 wt) and BRG1 knockout cells (BRG1 KO) at early, mid and late time points. Data are presented as the mean; n = 2. **d** Scaled abundance of CCND1 and CCND3 by proteome analysis in HCT116 (BRG1 wt) and BRG1 knockout cells (BRG1 KO) at early time points. Data are presented as the mean; n = 2.

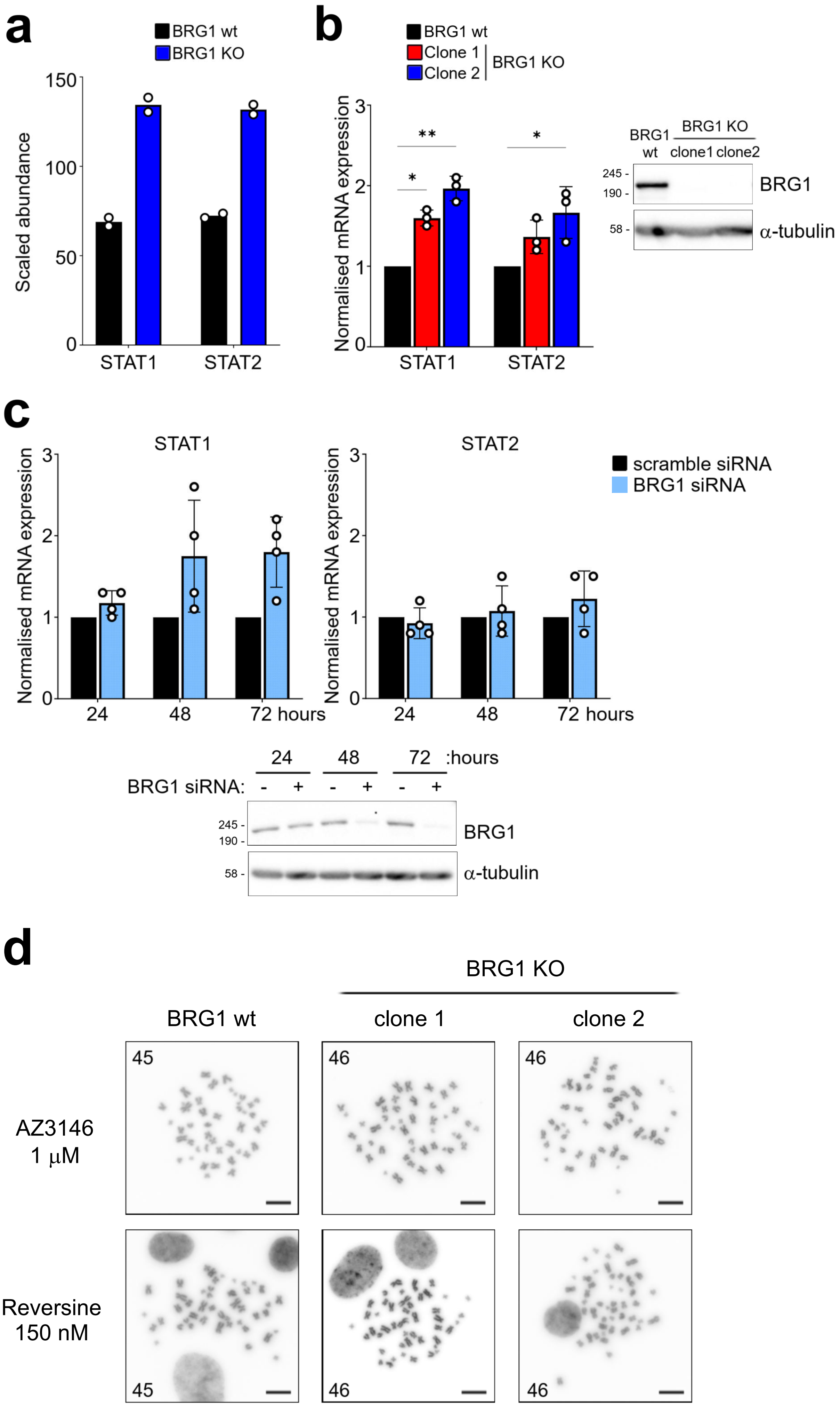
a
BRG1 wt
BRG1 KO
Scaled abundance
150
100
50
0
STAT1
STAT2
b
BRG1 wt
Clone 1
Clone 2
BRG1 KO
Normalised mRNA expression
3
2
1
0
**
*
*
STAT1
STAT2
BRG1 wt
BRG1 KO
clone1 clone2
245 -
190 -
58 -
BRG1
α-tubulin
c
STAT1
STAT2
Normalised mRNA expression
scramble siRNA
BRG1 siRNA
24
48
72 hours
24 48 72 :hours
BRG1 siRNA: - + - + - +
245 -
190 -
58 -
BRG1
α-tubulin
d
BRG1 KO
BRG1 wt
clone 1
clone 2
AZ3146
1 μM
Reversine
150 nM
45
46
46
45
46
46

Fig. S6 STAT1 and STAT2 mRNA and protein levels are generally upregulated when BRG1 is deficient. **a** Scaled abundance of STAT1 and STAT2 by proteome analysis in HCT116 and BRG1-deficient cells (clone 2, early time point). Data are presented as the mean; n = 2. **b** mRNA expression of *STAT1* and *STAT2* in HCT116 and the two BRG1 KO clones. The values have been normalised to GAPDH expression. Data are presented as the mean ±SD; n = 3. The p-value was calculated with two-way ANOVA-Dunnet. * $p < 0.05$, ** $p < 0.01$. Inset panel: Western blot analysis of BRG1. **c** mRNA expression of *STAT1* and *STAT2* in HCT116 at the indicated time points following siRNA treatment with a scramble control or siBRG1. The values have been normalised to GAPDH expression. Data are presented as the mean ±SD; n = 4. Lower panel: Western blot analysis of siRNA treated cells. α-tubulin was used as loading control. **d** Representative metaphase spreads prepared from the indicated cells following treatment with either AZ3146 or Reversine. The number of chromosomes in each spread is indicated. Scale bar = 10 μm.

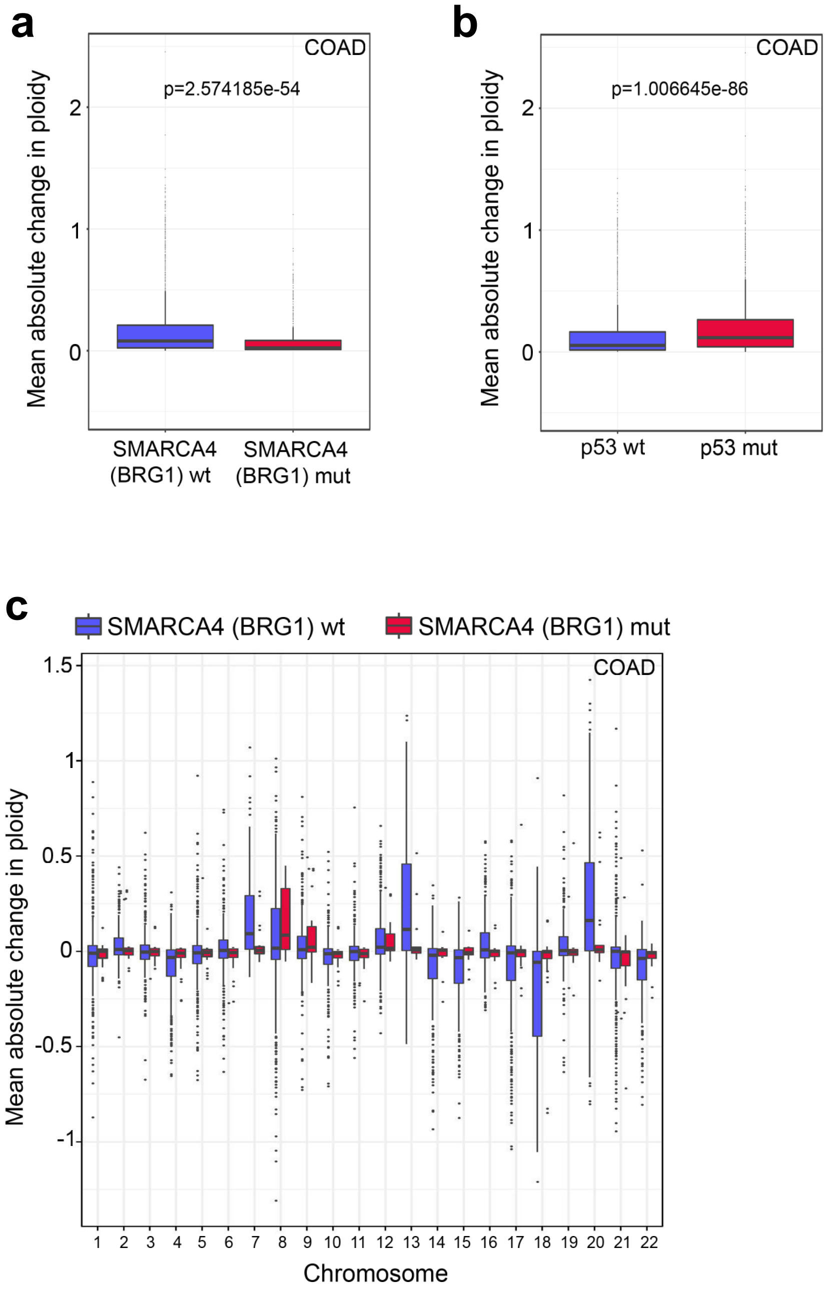
a
COAD
p=2.574185e-54
Mean absolute change in ploidy
2
1
0
SMARCA4 (BRG1) wt
SMARCA4 (BRG1) mut
b
COAD
p=1.006645e-86
Mean absolute change in ploidy
p53 wt
p53 mut
c
SMARCA4 (BRG1) wt
SMARCA4 (BRG1) mut
COAD
1.5
1
0.5
0
-0.5
-1
Mean absolute change in ploidy
1 2 3 4 5 6 7 8 9 10 11 12 13 14 15 16 17 18 19 20 21 22
Chromosome

Fig. S7 Aneuploidy scores in in Colon Adenocarcinoma (COAD)

a Colon Adenocarcinoma (COAD) samples that have mutations in the gene encoding BRG1 (SMARCA4) show lower aneuploidy scores than samples with no mutations in BRG1. TP53 mutant samples were excluded from the analysis to avoid confounding effects. The mean absolute change in aneuploidy was calculated as a deviation from diploid (zero), student t-test p=2.574e-54. **b** TP53 mutant samples were analysed separately as a point of comparison, student t-test p=1.006e-86. **c** Frequent loss of chromosome 18 in colorectal adenocarcinoma (COAD) is not apparent in samples with BRG1 mutations. Copy number variation for each chromosome in COAD samples analysed according to BRG1 (SMARCA4) mutational status. Positive aneuploidy score indicates chromosome gains and negative aneuploidy score indicates chromosome loss.
