# Supplementary material for: Aneuploidy tolerance caused by BRG1 loss allows chromosome gains and recovery of fitness
Source: Nat Commun. 2022 Apr 1;13:1731. doi: 10.1038/s41467-022-29420-3 (PMC8975814; doi:10.1038/s41467-022-29420-3)
Supplement: Supplementary file 3 — Reporting Summary [file 41467_2022_29420_MOESM3_ESM.pdf]

# nature portfolio

Corresponding author(s): J A Downs

Last updated by author(s): Mar 10, 2022

# Reporting Summary

Nature Portfolio wishes to improve the reproducibility of the work that we publish. This form provides structure for consistency and transparency in reporting. For further information on Nature Portfolio policies, see our Editorial Policies and the Editorial Policy Checklist.

## Statistics

For all statistical analyses, confirm that the following items are present in the figure legend, table legend, main text, or Methods section.

| n/a | Confirmed | |
|---|---|---|
| ☐ | ☒ | The exact sample size (*n*) for each experimental group/condition, given as a discrete number and unit of measurement |
| ☐ | ☒ | A statement on whether measurements were taken from distinct samples or whether the same sample was measured repeatedly |
| ☐ | ☒ | The statistical test(s) used AND whether they are one- or two-sided<br>*Only common tests should be described solely by name; describe more complex techniques in the Methods section.* |
| ☐ | ☐ | A description of all covariates tested |
| ☐ | ☒ | A description of any assumptions or corrections, such as tests of normality and adjustment for multiple comparisons |
| ☐ | ☒ | A full description of the statistical parameters including central tendency (e.g. means) or other basic estimates (e.g. regression coefficient) AND variation (e.g. standard deviation) or associated estimates of uncertainty (e.g. confidence intervals) |
| ☐ | ☒ | For null hypothesis testing, the test statistic (e.g. *F*, *t*, *r*) with confidence intervals, effect sizes, degrees of freedom and *P* value noted<br>*Give P values as exact values whenever suitable.* |
| ☒ | ☐ | For Bayesian analysis, information on the choice of priors and Markov chain Monte Carlo settings |
| ☒ | ☐ | For hierarchical and complex designs, identification of the appropriate level for tests and full reporting of outcomes |
| ☐ | ☒ | Estimates of effect sizes (e.g. Cohen's *d*, Pearson's *r*), indicating how they were calculated |

*Our web collection on statistics for biologists contains articles on many of the points above.*

## Software and code

Policy information about availability of computer code

| | |
|---|---|
| Data collection | Sequest HT within Proteome Discoverer2.2, BD FACSDiva 9.0, Slidebook6 |
| Data analysis | FlowJo v10.1, GraphPad Prism 9.3.0, Fiji/ImageJ 1.53c, Perseus 1.6, RStudio 3.6.1, Phantasus v1.11.0, ggplot2 |

For manuscripts utilizing custom algorithms or software that are central to the research but not yet described in published literature, software must be made available to editors and reviewers. We strongly encourage code deposition in a community repository (e.g. GitHub). See the Nature Portfolio guidelines for submitting code & software for further information.

## Data

Policy information about availability of data

All manuscripts must include a data availability statement. This statement should provide the following information, where applicable:

- Accession codes, unique identifiers, or web links for publicly available datasets
- A description of any restrictions on data availability
- For clinical datasets or third party data, please ensure that the statement adheres to our policy

We have provided a data availability statement with an accession code for the proteomics data (deposited to PRIDE) and with reference to the Source Data file.

# Field-specific reporting

Please select the one below that is the best fit for your research. If you are not sure, read the appropriate sections before making your selection.

☒ Life sciences ☐ Behavioural & social sciences ☐ Ecological, evolutionary & environmental sciences

For a reference copy of the document with all sections, see nature.com/documents/nr-reporting-summary-flat.pdf

# Life sciences study design

All studies must disclose on these points even when the disclosure is negative.

| | |
|---|---|
| Sample size | Sample sizes were chosen according to standard practice in the field. A minimum of three biological repeats for cell biology assays was used to allow statistical analysis of variability. When performing FACS analysis, a minimum of 10,000 events per sample was captured to minimise sampling errors. Three technical replicates per biological replicate were chosen for the RT-PCR assays to assess and control for assay variability. The number of metaphase spreads counted per sample was a function of the number of mitotic cells in the population. Bioinformatic analysis of cancer genomics (mRNA, mutations, copy number) made use of all publicly available data, which therefore defined the sample size. |
| Data exclusions | No data exclusions. |
| Replication | Reproducibility was tested using biological replicates for cell biology experiments and all experiments in the main figures were performed a minimum of three independent times.  Principle component analysis was used to assess the reproducibility of the proteomic data.  In addition, varying doses, time points and drugs were used to interrogate reproducibility (with a minimum of three indepedent experiments per condition), and results were consistent. |
| Randomization | Randomization was not relevant to this study because samples were not assigned to groups in the experimental design used here. |
| Blinding | Blinding was not used in this study because the majority of assays did not rely on subjective analysis that would be biased by the identification of samples (e.g. FACS profiles, CGH, RT-PCR and proteomic analysis). Where readouts relied on subjective assessment (e.g. microscopy), the morphology of the cells was altered in the KO, so identifiable even if blinding was used. |

# Reporting for specific materials, systems and methods

We require information from authors about some types of materials, experimental systems and methods used in many studies. Here, indicate whether each material, system or method listed is relevant to your study. If you are not sure if a list item applies to your research, read the appropriate section before selecting a response.

## Materials & experimental systems

| n/a | Involved in the study | |
|---|---|---|
| ☐ | ☒ | Antibodies |
| ☐ | ☒ | Eukaryotic cell lines |
| ☒ | ☐ | Palaeontology and archaeology |
| ☒ | ☐ | Animals and other organisms |
| ☒ | ☐ | Human research participants |
| ☒ | ☐ | Clinical data |
| ☒ | ☐ | Dual use research of concern |

## Methods

| n/a | Involved in the study | |
|---|---|---|
| ☒ | ☐ | ChIP-seq |
| ☐ | ☒ | Flow cytometry |
| ☒ | ☐ | MRI-based neuroimaging |

## Antibodies

| | |
|---|---|
| Antibodies used | Brg-1 (G-7), Santa Cruz Biotechnology, sc-17796, Lot:H1314<br>alpha-tubulin {DMI A), Abcam, Ab7291, Lot: GR310199-2<br>p21 Waf1/Cip1 (12D1), Cell signalling, 2947S, Lot:11<br>INO80, Bethyl, A303-371A<br>PICH, Millipore, 04-1540<br>Rabbit anti-Mouse HRP, DAKO, P0260<br>Goat anti-Rabbit HRP, DAKO, P0448 |
| Validation | Validation of primary antibodies:<br>1. BRG1 antibody: https://www.scbt.com/p/brg-1-antibody-h-10<br>Specific for an epitope mapping between amino acids 115-149, recognises human, mouse and rat according to manufacturer. In our lab, validated using siRNA of BRG1 and IP followed by Mass Spec.<br>2. Alpha-tubulin: https://www.abcam.com/alpha-Tubulin-antibody-DM1A-Loading-Control-ab7291.html<br>Recognises human, rat and mouse according to manufacturer.<br>3. p21 antibody: https://www.scbt.com/p/p21-antibody-f-8 |

Specific for aa124-164 of human p21, recognises human, mouse and rat according to manufacturer.
4. INO80 antibody: https://www.thermofisher.com/antibody/product/INO80-Antibody-Polyclonal/A303-371A
Recognises human INO80 according to manufacturer. Validated using siRNA of INO80 to show specificity of signal in our lab.
5. PICH antibody: https://www.merckmillipore.com/GB/en/product/Anti-PICH-Antibody-clone-142-26-3,MM_NF-04-1540
Recognises human PICH, validated in WB, IP and ICC according to manufacturer.

## Eukaryotic cell lines

Policy information about cell lines

| | |
|---|---|
| Cell line source(s) | HCT116 (ATCC) |
| Authentication | Validation using cell morphology, proliferation characteristics and CGH-array analysis. |
| Mycoplasma contamination | All cell lines were negative for mycoplasma contamination. |
| Commonly misidentified lines (See ICLAC register) | No commonly misidentified cell lines were used. |

## Flow Cytometry

### Plots

Confirm that:

☐ The axis labels state the marker and fluorochrome used (e.g. CD4-FITC).

☐ The axis scales are clearly visible. Include numbers along axes only for bottom left plot of group (a 'group' is an analysis of identical markers).

☒ All plots are contour plots with outliers or pseudocolor plots.

☒ A numerical value for number of cells or percentage (with statistics) is provided.

### Methodology

| | |
|---|---|
| Sample preparation | Cell line fixed with 70% ethanol. |
| Instrument | BD LSR II Flow Cytometer (BD Biosciences) |
| Software | BD FACSDiva<br>Flowjo v10.1 |
| Cell population abundance | Around 10000 single cells were used for the cell cycle profile analysis. |
| Gating strategy | Single cell population was selected through preliminary FSC-A/SSC-A gating followed by PE TxRed-A/PE TxRed-W gating. |

☒ Tick this box to confirm that a figure exemplifying the gating strategy is provided in the Supplementary Information.
